# Association of radiological lung pattern and respiratory mechanics with potential for lung recruitment in patients with COVID–ARDS: a retrospective cohort study

**DOI:** 10.1186/s40001-022-00821-w

**Published:** 2022-10-01

**Authors:** Hans-Jörg Gillmann, Carolin Jung, Milan Speth, Jens Vogel-Claussen, Thomas Stueber

**Affiliations:** 1 Department of Anaesthesiology and Intensive Care Medicine, Hannover Medical School, Carl-Neuberg Strasse 1, 30625 Hannover, Germany; 2 Institute for Diagnostic and Interventional Radiology, Hannover Medical School, Hannover, Germany

**Keywords:** COVID-19 Phenotype, ARDS, Recruitability, Computed tomography, Recruitment-to-inflation ratio

## Abstract

**Background:**

The ventilatory management of COVID–ARDS is controversial, especially with regard to the different subtypes and associated PEEP titration. A higher PEEP may be beneficial only in patients with potential for lung recruitment. The assessment of lung recruitment may be guided by lung imaging, such as electric impedance tomography or recruitment computed tomography, but is complex and not established in routine clinical practice. Therefore, bedside identification of recruitable ARDS phenotypes can aid in PEEP titration in clinical settings.

**Methods:**

In this retrospective consecutive cohort study in 40 patients with moderate-to-severe COVID–ARDS, we assessed lung recruitment using the recruitment-to-inflation ratio (R/I) in moderate-to-severe COVID–ARDS. Evidence of recruitment (R/I ≥ 0.5) was compared between clinical and computed tomography data.

**Results:**

Of the included patients, 28 (70%) were classified as recruiters by the R/I. Lung recruitment was associated with higher compliance and was not associated with a consolidated lung pattern assessed using CT. Even in the tertile of patients with the highest compliance (37–70 ml/mbar), eight (73%) patients were classified as recruitable. Patients classified as recruitable presented a lower reticular lung pattern (2% vs. 6%, *p* = 0.032).

**Conclusions:**

Prediction of lung recruitment is difficult based on routine clinical data but may be improved by assessment of radiographic lung patterns. A bedside assessment of recruitment is necessary to guide clinical care. Even a high compliance may not rule out the potential for lung recruitment.

## Background

Since the beginning of the COVID-19 pandemia, a large number of critically ill patients with a clinical diagnosis of ARDS according to the Berlin definition have been treated in intensive care. As most virus-specific treatment options fail in clinical trials, great emphasis is placed on optimal basic critical care management. In the case of ARDS, this includes lung-protective ventilation using a low tidal volume, and treatment with appropriate levels of PEEP. However, there is an ongoing debate regarding the optimal ventilatory management of critically ill COVID-19 patients. Contradictory advice is aggravated by the fact that even in non-COVID–ARDS patients, accurate ventilatory management remains a matter of debate. Although studies indicate that higher PEEP is beneficial in moderate to severe ARDS as defined by the Berlin definition [1, 2], no single randomised controlled trial has shown that a single method of PEEP titration is beneficial. This may be due to the fact, that higher PEEP is only beneficial in ARDS patients with recruitable lungs, while it leads to overinflation and damage of the non-recruitable lung. Several phenotypes, such as focal or non-focal phenotypes in Non-COVID ARDS have been proposed to guide ventilatory management and PEEP settings. However, a randomised controlled trial that tailored mechanical ventilation to a focal or nonfocal phenotype failed to improve the outcome [3].

Furthermore, there is an ongoing debate on whether distinct COVID–ARDS-specific phenotypes exist, and if those phenotypes can guide ventilatory management [4]. Gattinoni described L- and H-phenotypes according to the compliance of the COVID–ARDS lung. According to these phenotypes, the authors suggested treatment with lower PEEP and higher tidal volumes (8–9 ml/kg) in patients with high compliance and lower recruitability (L-type) as opposed to a higher PEEP, and lower tidal volume (4–6 ml/kg) in patients with low compliance and higher recruitability (H-type) [4]. From a radiologic perspective, there is a huge variation in lung morphology in COVID–ARDS, with varying proportions of ground-glass opacities, consolidation, or reticular patterns.

Different studies have shown varying proportions of recruitable patients with COVID–ARDS, probably because of assessment at different disease timepoints [5–7]. To date, there is insufficient data regarding different COVID–ARDS phenotypes, lung mechanics, or radiologic lung morphology and their association with recruitability. In this retrospective study, we, therefore, aimed to investigate the association of these clinical and radiologic characteristics with lung recruitability assessed by the recruitment-to-inflation ratio in a cohort of COVID patients with moderate to severe ARDS.

## Methods

### Study design and population

Ethical approval for this study (Ethical Committee N° 9949_BO_K_2021) was provided by the Hannover Medical School Ethics Committee, Hannover, Germany (Chairperson Prof. B. Schmidt) on 12th of August 2021. The requirement for informed consent was waived because of the retrospective design of the study. We included COVID-19 patients admitted to the anaesthesia intensive care unit (ICU, Department of Anesthesia and Intensive Care Medicine, Hannover Medical School) from October 2020 until August 2021. The anaesthesia intensive care unit is one of 6 independent surgical intensive care units at Hannover Medical School as a tertiary referral hospital and one of 2 intensive care units dedicated to care for COVID-19 patients during the pandemic surge. A dedicated SOP (Standard Operating Procedure) for the management of COVID-19 patients continuously adopted the AWMF (Arbeitsgemeinschaft der Wissenschaftlichen, Medizinischen Fachgesellschaften e.V.) S3 guideline recommendations [8]. Ventilator management, including the choice of PEEP, was performed according to the treating physician. CT imaging and assessment of recruitment to inflation ratio were performed as a routine part of clinical ARDS evaluation [9]. Hannover Medical School is a tertiary centre and most of the patients had severe ARDS with ECMO therapy.

### Inclusion and exclusion criteria

This study was designed to investigate the predictors of recruitment in mechanically ventilated COVID–ARDS patients presenting with moderate-to-severe illness at intensive care unit admission. Therefore, we included patients (1) with confirmed COVID-19 disease by polymerase chain reaction testing, (2) with an ARDS diagnosis based on the Berlin definition [2], (3) with documentation of treatment within the standard patient data management system of the ICU, and (4) aged 18 years or older. Patients were excluded if (1) they were admitted repeatedly, (2) the recruitment-to-inflation ratio was not documented, (3) no computed tomography (CT) of the thorax was available, (4) patients were treated for main diagnoses other than COVID-19, or (5) patients were diagnosed with advanced COPD.

### Recruitment-to-inflation ratio

The R/I was assessed as previously described with a PEEP high of 15 mbar and a PEEP low of 5 mbar or with the value of airway opening pressure (AOP) if AOP was > 5 mbar [9]. Before the R/I ratio was assessed, a low-flow inflation manoeuvre was performed to measure airway opening pressure (AOP). We excluded all patients with an AOP higher than 10 mbar, because a PEEP of at least 5 mbar higher than the AOP is recommended for the assessment of the R/I ratio. R/l measurements were performed in sedated and paralysed patients ventilated with Hamilton C6 order Evita 4. Before R/I ratio assessment, PEEP was set at 15 mbar for at least 20 min to allow stabilization of hemodynamics and pulmonary mechanics as per local standard ARDS treatment protocol. A tidal volume of 5–6 ml/kg was used. The set tidal volume (VTset), exhaled volume during the single-breath release from PEEP high to PEEP low (Vtrelease), exhaled volume at high (Vthigh) and low (Vtlow) PEEP, and plateau pressure (Pplat) were recorded. The R/I ratio was calculated as follows [9]:

$${{({\text{Vtrelease}}\, - {\text{Vthigh}})} \mathord{\left/ {\vphantom {{({\text{Vtrelease}}\, - {\text{Vthigh}})} {{\text{Vtset}}\, \times }}} \right. \kern-\nulldelimiterspace} {{\text{Vtset}}\, \times }}\,{{\left( {{\text{Pplat}} - {\text{PEEPlow}}} \right)} \mathord{\left/ {\vphantom {{\left( {{\text{Pplat}} - {\text{PEEPlow}}} \right)} {\left( {{\text{PEEPhigh}} - {\text{PEEPlow}}} \right)}}} \right. \kern-\nulldelimiterspace} {\left( {{\text{PEEPhigh}} - {\text{PEEPlow}}} \right)}} - 1$$

### Data collection

Treatment in the study ICU was documented using the patient data management system (PDMS) (Medisite GmbH, Hannover, Germany). Patient data (anthropometric and baseline data, medical history, vital parameters, and ICU data) were extracted manually into a Microsoft Excel (Microsoft Corporation, Redmond, WA, USA) study worksheet, with the data being stored on a secured intrahospital server. After completion and cross-checking of the worksheets, patient data were deleted. The remaining data were exported to SPSS (SPSS, Chicago, IL, USA) for anonymised data analyses. Computed tomography data were processed using ‘AVIEW’ (Coreline Soft, Republic of Korea), an automated segmentation and quantitative lung texture analysis software, and cross-validated by a radiologist blinded to clinical and bedside data.

### Study endpoints and main outcome measures

Patients were classified as recruiters or non-recruiters based on the measurement of the recruitment to inflation ratio (RI < 0.5 vs. RI ≥ 0.5) [9]. Patients with an airway occlusion pressure of ≥ 10 mbar were excluded, because a valid RI manoeuvre was not possible. Regarding computed tomography data, both the entire lung volume and lung tissues with consolidated, reticular, ground-glass opacities, and normal radiographic patterns were quantified in absolute numbers, and the tissues were related to the entire volume and quantified in relative numbers. Patients with an intact pregnancy were excluded because of altered ventilation mechanics due to pregnancy and obvious relative contraindications to CT examination. As we aimed to identify the predictors of lung recruitment in COVID–ARDS, patients were dichotomised based on RI into recruiter and non-recruiter groups. Laboratory measurements were recorded on the day of the RI manoeuvre. In our centre, IL-2 and IL-6 were measured in all COVID–ARDS patients since the beginning of the COVID-19 pandemia [10]. If there were no measurements of IL-2 and IL-6 on the RI manoeuvre day, the last valid measurement was recorded. If there was more than one measurement during the day, the mean value was calculated.

Secondary outcome measures were added to characterise illness severity and early outcomes among the included patients. Therefore, we recorded (1) the incidence of acute kidney injury (AKI), (2) AKI requiring dialysis, and (3) ICU mortality.

### Statistical analysis

Because of the exploratory design of this study, we aimed to include the largest possible sample size from the ICU database. We extended the maximum recruitment interval to October 2020, starting with the systematic admission of COVID-19 patients to the study ICU.

Data are presented as medians with their respective interquartile ranges (IQRs) or as means and 95% confidence intervals, as appropriate. Data were tested non-parametrically using Mann–Whitney *U* tests, because computed tomography and clinical data were distributed non-normally for methodological reasons. Data were analysed using SPSS (SPSS, Chicago, IL, USA) and MedCalc (MedCalc Software, Ostende, Belgium).

## Results

### Patient characteristics

**Fig. 1:**
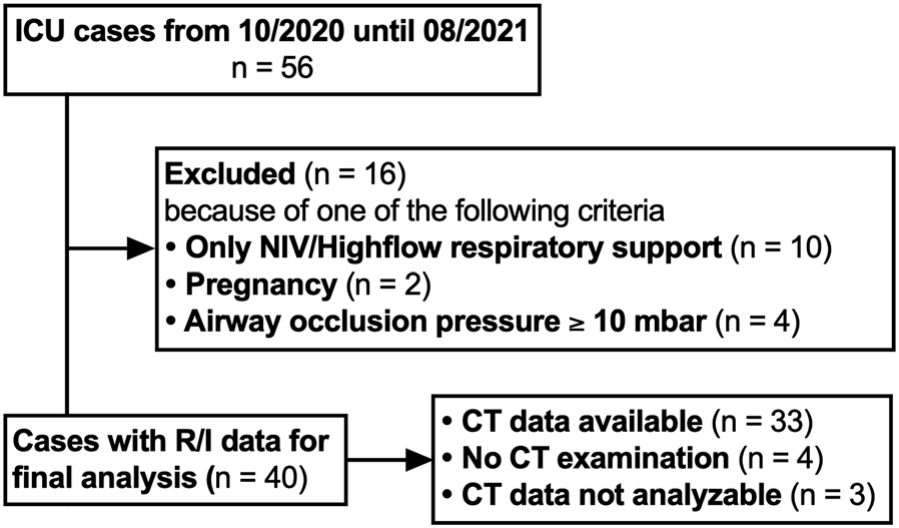
Patient flow chart. *CT* computed tomography, *NIV* non-invasive ventilation; *R/I* recruitment to inflation ratio. Four patients in the final analysis cohort did not have CT data, whereas three CT data sets were not evaluable by the used algorithm. This resulted in 33 patients with a complete data set for all study variables

During the study period, 56 patients with a diagnosis of COVID–ARDS confirmed by a positive SARS-CoV-2 test were admitted to the ICU, resulting in 40 patients meeting the study inclusion and exclusion criteria (Fig. 1). Of these, seven patients had unavailable CT data (four patients without computed chest tomography; three patients with computed tomography images not analyzable by the used algorithm). Five patients (12.5%) were included with a CT data set acquired from the assigned hospitals. We were able to include 40 COVID–ARDS patients with a measured recruitment to inflation ratio. Thirty-three patients with analysable CT data were included. In 32 patients, the recruitment-to-inflation ratio was assessed on the day of computed tomography imaging, while in one patient, the R/I ratio was collected 1 day after CT (CT recorded in the referring hospital).

### COVID-19 stage of disease at ICU admission

The baseline characteristics of patients did not differ between recruiters and non-recruiters (Table 1). Most patients were admitted to peripheral hospitals for vvECMO evaluation or had established rescue vvECMO treatment. Patients were hospitalized 6 days (IQR: 1–11 days) prior to study ICU admission with 1 day (IQR: 0–4 days) of invasive ventilation in median. We did not detect a relevant difference in recruiters and non-recruiters regarding prior length of hospital treatment (RI < 0.5 vs. ≥ 0.5: 6.5 days vs. 6.2 days; *p* = 0.896) duration of mechanical ventilation (RI < 0.5 vs. ≥ 0.5: 2.8 days vs. 2.4 days; *p* = 0.965) or viral load (RI < 0.5 vs. ≥ 0.5: 31 ct vs. 32 ct; *p* = 0.965). RI was measured as the median on ICU day 1 (IQR: 0–5 days). The day of RI assessment also did not differ between the two compared patients groups (RI < 0.5, ≥ 0.5, 3.0, and 3.8; *p* = 0.299). Seventeen patients (42.5%) were admitted for vvECMO. Thirty (75%) patients were treated with vvECMO support during ICU stay. The inflammatory parameters (Table 2) and incidence of clinical endpoints (Table 3) in the two patient groups were comparable.

**Table 1:** Baseline characteristics of the patients

Quantitative parameters	Total (*n* = 40)	RI < 0.5 (*n* = 12)	RI ≥ 0.5 (*n* = 28)	*p* value
	Median (IQR)	Median (IQR)	Median (IQR)	
Age (year)	58 (47 to 65)	57 (52 to 64)	59 (43 to 67)	0.896
Weight (kg)	98 (90 to 110)	92 (100 to 110)	95 (86 to 114)	0.475
Height (cm)	179 (170 to 183)	170 (175 to 180)	180 (166 to 185)	0.224
SAPS-II at admission	43 (36 to 52)	39 (36 to 53)	43 (34 to 52)	0.899
SARS-CoV-2 PCR (ct)	31 (28 to 37)	31 (28 to 36)	32 (27 to 37)	0.965
Days of invasive ventilation before RI (d)	2 (1 to 11)	4 (1 to 10)	2 (1 to 13)	0.984
PEEP before RI (mbar)	14 (12 to 15)	14 (13 to 15)	15 (12 to 15)	0.942
Compliance before RI (ml mbar^−1^)	30 (21 to 40)	17 (13 to 36)	32 (26 to 42)	0.005
Initial PF ratio (prior to vvECMO)	82 (68 to 116)	75 (60 to 88)	104 (72 to 122)	0.058

Qualitative parameters	Total (*n* = 40)	RI < 0.5 (*n* = 12)	RI ≥ 0.5 (*n* = 28)	*p* value
	% (*n*)	% (*n*)	% (*n*)	
Gender male	75.0 (30)	66.7 (8)	78.6 (22)	0.671
vvECMO therapy	75.0 (30)	91.7 (11)	67.9 (19)	0.247

ct, real-time polymerase chain reaction cycle time. Baseline characteristics between the two patient groups with regard to the presented parameters did not differ clinically relevant. *p* value: Mann–Whitney *U* test

*IQR* interquartile range. *SAPS* simplified acute physiology score, *SARS-CoV-2* severe acute respiratory syndrome coronavirus 2, *PCR* polymerase chain reaction, *PEEP* positive end-expiratory pressure, *RI* recruitment to inflation ratio, *vvECMO* veno-venous extracorporeal membrane oxygenation

**Table 2:** Inflammatory parameters

Quantitative parameters	Total (*n* = 40)	RI < 0.5 (*n* = 12)	RI ≥ 0.5 (*n* = 28)	*p* value
	Median (IQR)	Median (IQR)	Median (IQR)	
CRP (mg l^−1^)	184 (97–277)	201 (126–297)	175 (79–269)	0.512
d-Dimers (mg l^−1^)	3.3 (1.5–15.1)	4.4 (1.6–17.1)	2.9 (1.5–13.8)	0.738
Ferritin ECLIA (µg l^−1^)	1122 (706–2580)	1289 (383–3153)	1122 (733–2441)	0.896
IL-2 (kU l^−1^)	1549 (937–2543)	1578 (984–3632)	1549 (883–2501)	0.986
IL-6 (ng l^−1^)	92 (39–523)	184 (48–849)	75 (34–489)	0.405
Leukocytes (× 10^9^ l^−1^)	13.5 (11.0–18.3)	13.1 (11.9–14.8)	14.6 (10.5–19.4)	0.422
Procalcitonin (µg l^−1^)	0.8 (0.3–3.0)	1.3 (0.3–13.1)	0.7 (0.3–2.4)	0.422

*p* value: Mann–Whitney *U* test. Measurements were recorded on the day of the RI manoeuvre. If there were no measurements for IL-2 and IL-6 at the RI manoeuvre day, last valid measurements were recorded. If there was more than one measurement during the day, the mean value was calculated

*IQR* interquartile range, *CRP* C-reactive protein, *ECLIA* electrochemiluminescence immunoassay, *IL* interleukin, *RI* recruitment to inflation ratio, *SAPS* simplified acute physiology score

**Table 3:** Clinical endpoints

Quantitative parameters	Total (*n* = 40)	RI < 0.5 (*n* = 12)	RI ≥ 0.5 (*n* = 28)	*p* value
	Median (IQR)	Median (IQR)	Median (IQR)	
Duration of invasive ventilation (h)	423 (339–870)	515 (340–1054)	419 (334–711)	0.568
ICU length of stay (d)	20 (16–41)	23 (13–44)	19 (16–35)	0.899
vvECMO duration (d)	17 (9–28)	14 (7–28)	18 (10–31)	0.582

Qualitative parameters	Total (*n* = 40)	RI < 0.5 (*n* = 12)	RI ≥ 0.5 (*n* = 28)	*p* value
	% (*n*)	% (*n*)	% (*n*)	
Acute kidney injury	70 (28)	67 (8)	71 (20)	0.730
Dialysis required	40 (16)	50 (6)	36 (10)	0.443
Mortality	38 (15)	50 (6)	32 (9)	0.389

*p* value: Mann–Whitney *U* test. RI was not associated with the recorded clinical outcomes

*IQR* interquartile range, *RI* recruitment to inflation ratio

### Higher recruitability in patients with increased compliance

**Fig. 2:**
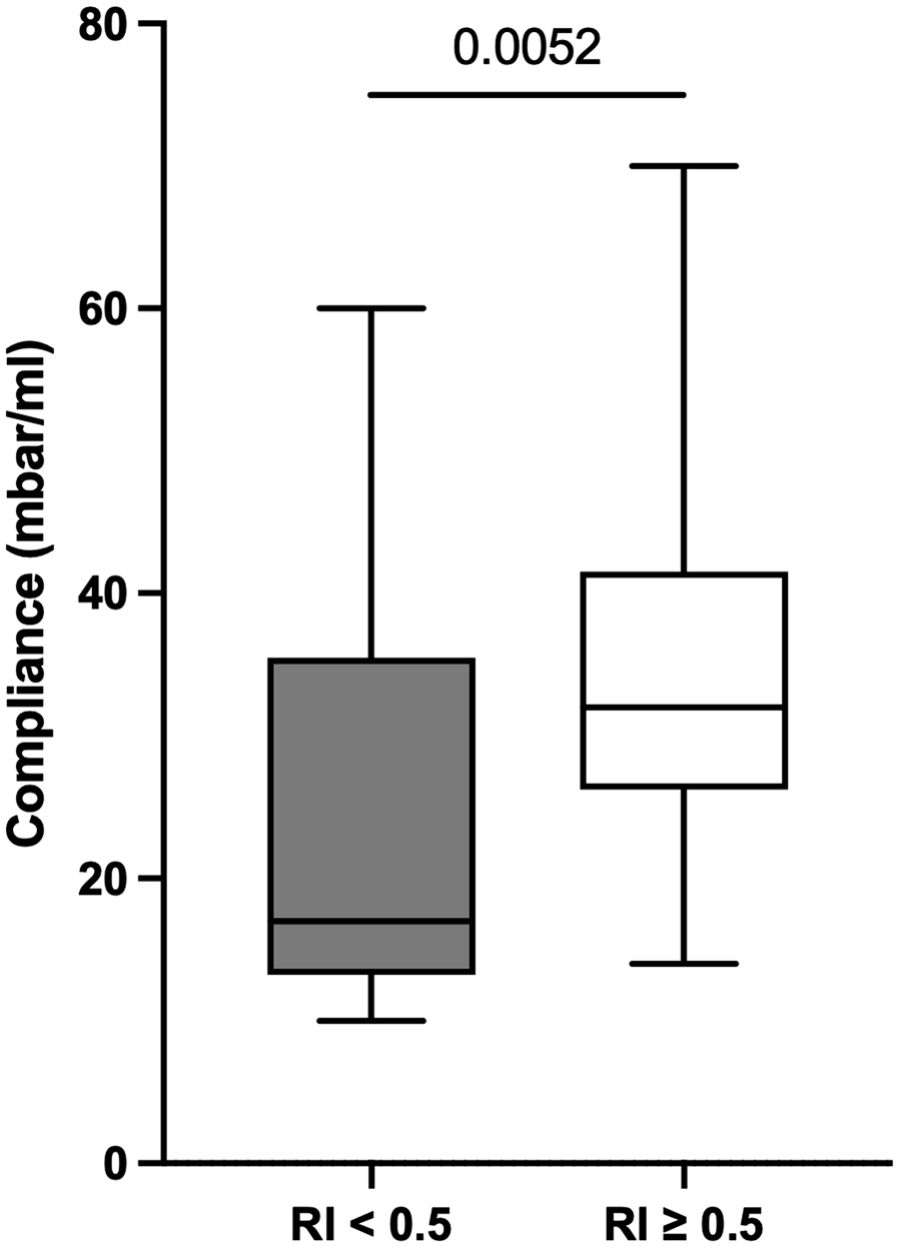
Compliance and recruitability. RI, recruitment to inflation ratio. *p* value: Mann–Whitney *U* test. Recruitability (RI ≥ 0.5) was associated with an increased pulmonal compliance prior to RI manoeuvre

**Fig. 3:**
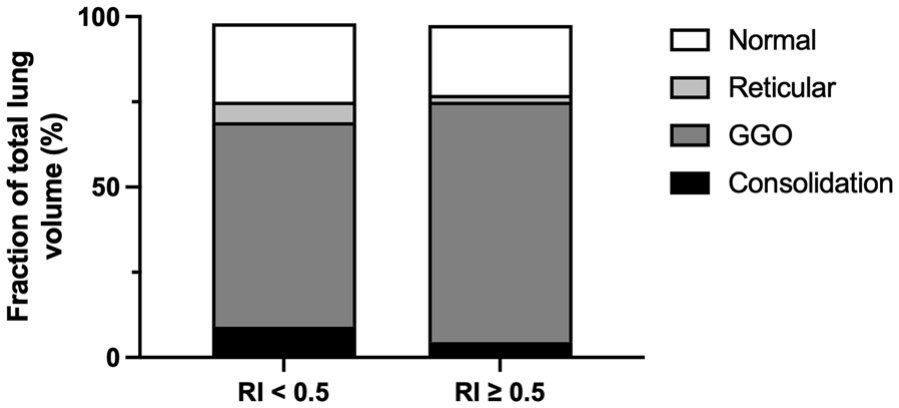
Computed tomography data and recruitability. GGO, ground-glass opacity; RI, recruitment to inflation ratio. The diagnostic CT algorithm automatically differentiated between lung texture with normal, reticular, ground-glass opacified or consolidated tissue (data for lung texture with emphysema or honeycomb characteristics are not shown and explain the gap to 100%). Patients classified as non-recruiters presented with an increased fraction of lung tissue with reticular texture (6% (IQR: 3–8%) vs. 2% (0–3%); *p* = 0.032). Fractions of lung volume with normal, GGO or consolidated pattern did not differ between both groups

Based on the RI manoeuvre (cutoff: RI 0.5), 12 patients were classified as non-recruiters, and 28 (70%) were classified as recruiters. Recruiters presented with higher static compliance than non-recruiters (median: 32 ml/mbar [IQR: 26-42 ml/mbar] vs. 17 ml/mbar [IQR: 13-36 ml/mbar]; *p* = 0.0052; Fig. 2). Within the tertile of patients with the highest compliance (compliance 37–70 ml/mbar), 8 of the 11 patients were classified as recruiters (RI ≥ 0.5). CT data showed comparable results for non-recruiters and recruiters with regard to diseased lung tissue (texture with relevant lung pathology 76% [IQR: 69–89%] vs. 80% [IQR: 67–95%]; *p* = 0.796). The only CT parameter with a statistically significant difference between non-recruiters and recruiters was the fraction of lung tissue with a reticular texture (6% [IQR: 3–8%] vs. 2% [0–3%]; *p* = 0.032; Fig. 3 and Table 4). ROC analysis for discrimination between recruiters and non-recruiters showed a statistically significant accuracy for the fraction of lung tissue with reticular texture (AUC 0.75 (95% CI 0.55–0.94), *p* = 0.032), but not for compliance (AUC 0.71 (95% CI 0.46–0.97), *p* = 0.106).

**Table 4:** Computed tomography data

Quantitative parameters	Total (*n* = 33)	RI < 0.5 (*n* = 9)	RI ≥ 0.5 (*n* = 24)	*p* value
	Median (IQR)	Median (IQR)	Median (IQR)	
Normal (%)	22 (9–32)	23 (11–29)	21 (4–32)	0.796
Reticular (%)	2 (1–7)	6 (3–8)	2 (0–3)	0.032
GGO (%)	69 (53–79)	60 (48–79)	71 (56–85)	0.290
Consolidation (%)	5 (2–9)	9 (3–19)	5 (2–9)	0.179

*p* value: Mann–Whitney *U* test. Numeric computed tomography data. Percentages do not add to 100, because honeycomb and emphysema pattern fractions are not presented because of their clinical irrelevance in this cohort. Recruitment was associated with a decreased fraction of lung tissue with reticular pattern

*GGO* ground glass opacity, *IQR* interquartile range, *RI* recruitment to inflation ratio

## Discussion

Our study showed that in a cohort with severe lung failure due to SARS-COV-2 most of the included patients were recruitable. The amount of reticular lung CT pattern was lower in patients classified as recruitable but with only marginal predictive significance. Contrary to other data from COVID– and Non-COVID–ARDS [11], higher compliance was associated with recruitment. Even among patients with high compliance, a high proportion is recruitable. Inflammatory markers were not associated with lung recruitment. The outcome did not differ significantly between recruiters and non-recruiters; however, the cohort was too small to draw reliable conclusions.

PEEP titration is an important component of mechanical ventilation in patients with acute respiratory failure. However, there is insufficient data regarding the method of PEEP titration. Considering the pathophysiology, higher PEEP may only be beneficial for recruiters. Nevertheless, although there are methods to assess recruitment at the bedside, such as quantitative analysis of recruitment by computed tomography [12], electric impedance tomography [13] or the recruitment-to-inflation ratio, these methods are time-consuming or complex, and therefore seldom used at the bedside. It would be an improvement for bedside clinicians if recruitment could be estimated from easily obtainable clinical data, such as respiratory mechanics, CT scans, or inflammatory biomarkers. For classic ARDS, latent class analysis identified clinical factors associated with recruitability [14], but COVID–ARDS is scarce. Therefore, the present study was conducted.

Our study showed that 70% of the patients were recruitable, which is comparable to the cohort of Grieco et al. [5]. Other studies have shown variable fractions of recruitable patients ranging from 20 to 60% [7, 15, 16]. This may be due to the different study populations with regard to disease severity and the timepoint of the disease. Our study evaluated lung recruitability in a cohort with a severe degree of COVID-19 associated lung injury, emphasized by the low median P/F ratio and the high percentage of patients receiving ECMO-therapy [17]. At this stage of illness, the potential for recruitment may be rather high.

In a study of classic ARDS, radiological lung pattern and morphology predicted potential for recruitment [18]. In our study, the amount of consolidation and ground glass opacity were not associated with recruitability. This may be due to the different pathology of lung injury in COVID–ARDS compared to non-COVID–ARDS. In principle, consolidations can occur because of oedema or destroyed alveoli which may not be recruitable. Unfortunately, we did not differentiate the nature of consolidation using CT at lower and higher PEEP levels, which is a limitation of this study. In our study, a reticular lung pattern that may indicate lung scarring or fibrosis was associated with non-recruitment. The time course of pulmonary tissue alterations in COVID–ARDS is currently unknown; however, a reticular lung pattern may be associated with a later stage of the illness. Nevertheless, recruiters and non-recruiters in our study appeared to be in comparable stages of illness based on their prior hospital length of stay, duration of mechanical ventilation, and viral load at admission to our ICU. Thus, the amount of reticular lung texture may not only be associated with the stage of illness but also with illness severity and other currently unknown factors.

Higher patient compliance was associated with recruitment in the patient cohort. Two other studies in patients with COVID–ARDS showed no difference in PF ratio or compliance between recruitable and non-recruitable patients [6, 7]. This is in contrast to classic ARDS, in which patients with lower compliance and lower P/F ratio had a higher percentage of recruitable lungs [11]. This may be due to the complex pathophysiology of COVID–ARDS with direct lung damage, as well as endothelial and pulmonary vessel involvement. Therefore, the association between compliance, degree of lung parenchymal injury, degree of hypoxemia, and potential for recruitment may differ between COVID–ARDS and classic ARDS.

Although not statistically significant, our data point towards a higher mortality and incidence of dialysis in non-recruitable patients. Data from classical ARDS show conflicting results with regard to the association between recruitability and outcome [11, 19]. Because disease severity, mechanism of tissue injury and ventilatory management may influence the connection between recruitability and outcome, we believe that assessment of the potential for lung recruitment alone cannot reliably predict clinical outcome in ARDS or COVID–ARDS patients.

One limitation of our study is that the maximum PEEP was 15 mbar. Thus, we did not evaluate lung recruitment at higher airway pressures. We refrained from using higher PEEP levels in clinical practice because of the high percentage of macroscopic lung damage in COVID-19 patients and the unclear hemodynamic risk of high airway pressures. Another limitation is that we assessed the potential for lung recruitment using only the recruitment-to-inflation ratio and not lung imaging. Therefore, we could not detect the regional effects of the different PEEP levels. Because our cohort represents patients admitted to a single tertiary center for ECMO therapy or evaluation, the external validity of our findings may be limited. Given that computed tomography was not performed in all patients, this may also have introduced a bias by indication. Furthermore, the relatively small sample size limits the statistical power of our findings. We therefore consider our findings as hypothesis-generating that need confirmation in larger prospective multicentric studies.

## Conclusions

We could not identify a clinically useful association between lung recruitment and inflammatory markers, but potentially with the amount of reticular lung pattern quantified by CT. Due to the high variability of recruitable patients between different cohort studies, our findings strongly indicate that recruitment must be assessed individually for every patient with COVID–ARDS. Even patients with COVID–ARDS with higher compliance may have potential for lung recruitment.

## Data Availability

The data sets generated and/or analysed during the current study are not publicly available due to data protection regulations, but are available from the corresponding author on reasonable request.

## Abbreviations

AKI: Acute kidney injury
AOP: Airway opening pressure
ARDS: Acute respiratory distress syndrome
AUC: Area under the receiver operating characteristic curve
CT: Computed tomography
COVID-19: Coronavirus disease 2019
ICU: Intensive care unit
IQR: Interquartile range
PDMS: Patient data management system
PEEP: Positive end-exspiratory pressure
R/I: Recruitment to inflation ratio
vvECMO: Veno-venous extracorporeal membrane oxygenation

## References

[1] Briel M, Meade M, Mercat A, Brower RG, Talmor D, Walter SD, Slutsky AS, Pullenayegum E, Zhou Q, Cook D (2010). Higher vs lower positive end-expiratory pressure in patients with acute lung injury and acute respiratory distress syndrome: systematic review and meta-analysis. JAMA.

[2] Ranieri VM, Rubenfeld GD, Thompson BT, Ferguson ND, Caldwell E, Fan E, Camporota L, Slutsky AS (2012). Acute respiratory distress syndrome: the Berlin definition. JAMA.

[3] Constantin J-M, Monsel A, Blanchard F, Godet T (2019). Personalised mechanical ventilation in acute respiratory distress syndrome: the right idea with the wrong tools? —Authors’ reply. Lancet Respir Med.

[4] Gattinoni L, Chiumello D, Caironi P, Busana M, Romitti F, Brazzi L, Camporota L (2020). COVID-19 pneumonia: different respiratory treatments for different phenotypes?. Intensive Care Med.

[5] Grieco DL, Bongiovanni F, Chen L, Menga LS, Cutuli SL, Pintaudi G, Carelli S, Michi T, Torrini F, Lombardi G (2020). Respiratory physiology of COVID-19-induced respiratory failure compared to ARDS of other etiologies. Crit Care.

[6] Ball L, Robba C, Maiello L, Herrmann J, Gerard SE, Xin Y, Battaglini D, Brunetti I, Minetti G, Seitun S (2021). Computed tomography assessment of PEEP-induced alveolar recruitment in patients with severe COVID-19 pneumonia. Crit Care.

[7] Beloncle FM, Pavlovsky B, Desprez C, Fage N, Olivier P-Y, Asfar P, Richard J-C, Mercat A (2020). Recruitability and effect of PEEP in SARS-Cov-2-associated acute respiratory distress syndrome. Ann Intensive Care.

[8] Kluge S, Janssens U, Welte T, Weber-Carstens S, Schälte G, Spinner CD, Malin JJ, Gastmeier P, Langer F, Wepler M, et al. S3-Leitlinie - Empfehlungen zur stationären Therapie von Patienten mit COVID-19. AWMF. 2021. AWMF-Register-Nr. 113/001:1–89. 10.1055/a-1334-1925 33450783

[9] Chen L, Del Sorbo L, Grieco DL, Junhasavasdikul D, Rittayamai N, Soliman I, Sklar MC, Rauseo M, Ferguson ND, Fan E (2020). Potential for lung recruitment estimated by the recruitment-to-inflation ratio in acute respiratory distress syndrome. A clinical trial. Am J Respir Crit Care Med.

[10] Calfee CS, Delucchi K, Parsons PE, Thompson BT, Ware LB, Matthay MA (2014). Subphenotypes in acute respiratory distress syndrome: latent class analysis of data from two randomised controlled trials. Lancet Respir Med.

[11] Gattinoni L, Caironi P, Cressoni M, Chiumello D, Ranieri VM, Quintel M, Russo S, Patroniti N, Cornejo R, Bugedo G (2006). Lung recruitment in patients with the acute respiratory distress syndrome. N Engl J Med.

[12] Chiumello D, Formenti P, Coppola S (2019). Lung recruitment: what has computed tomography taught us in the last decade?. Ann Intensive Care.

[13] Mauri T, Mercat A, Grasselli G (2019). What’s new in electrical impedance tomography. Intensive Care Med.

[14] Wendel Garcia PD, Caccioppola A, Coppola S, Pozzi T, Ciabattoni A, Cenci S, Chiumello D (2021). Latent class analysis to predict intensive care outcomes in acute respiratory distress syndrome: a proposal of two pulmonary phenotypes. Crit Care.

[15] Haudebourg AF, Perier F, Tuffet S, de Prost N, Razazi K, Mekontso Dessap A, Carteaux G (2020). Respiratory mechanics of COVID-19- versus non-COVID-19-associated acute respiratory distress syndrome. Am J Respir Crit Care Med.

[16] Mauri T, Spinelli E, Scotti E, Colussi G, Basile MC, Crotti S, Tubiolo D, Tagliabue P, Zanella A, Grasselli G (2020). Potential for lung recruitment and ventilation-perfusion mismatch in patients with the acute respiratory distress syndrome from coronavirus disease 2019. Crit Care Med.

[17] Chiumello D, Bonifazi M, Pozzi T, Formenti P, Papa GFS, Zuanetti G, Coppola S (2021). Positive end-expiratory pressure in COVID-19 acute respiratory distress syndrome: the heterogeneous effects. Crit Care.

[18] Coppola S, Pozzi T, Gurgitano M, Liguori A, Duka E, Bichi F, Ciabattoni A, Chiumello D (2021). Radiological pattern in ARDS patients: partitioned respiratory mechanics, gas exchange and lung recruitability. Ann Intensive Care.

[19] Camporota L, Caricola EV, Bartolomeo N, Di Mussi R, Wyncoll DLA, Meadows CIS, Amado-Rodriguez L, Vasques F, Sanderson B, Glover GW (2019). Lung recruitability in severe acute respiratory distress syndrome requiring extracorporeal membrane oxygenation. Crit Care Med.

